# Phylogenetic analysis of *vp2* gene of the infectious bursal disease virus in South China during 2023

**DOI:** 10.3389/fvets.2025.1575407

**Published:** 2025-04-15

**Authors:** Kensi Zhu, Qi Wu, Mei Leng, Zhanxin Wang, Wencheng Lin

**Affiliations:** ^1^College of Animal Science, South China Agricultural University, Guangzhou, China; ^2^College of Veterinary Medicine, South China Agricultural University, Guangzhou, China; ^3^Wens Food Group Co., Ltd., Yunfu, China

**Keywords:** infectious bursal disease virus (IBDV), *vp2* gene, phylogenetic analysis, virus isolation, immunosuppression

## Abstract

Infectious Bursal Disease (IBD) is an acute, highly infectious, immunosuppressive disease caused by the infectious bursal disease virus (IBDV). To elucidate the prevalence of IBDV in southern China, a total of 60 tissues (including spleen and bursa) suspected of IBDV infection were collected from broiler chickens in 2023. In this study, a total of 31 IBDV strains were successfully isolated. The *vp2* gene sequences of these isolates were sequenced and analysed. The results showed that 8 of the isolates were identified as very virulent strains, 11 as classical strains and 12 as novel variants. The nucleotide sequence identity among the isolates ranged from 90.7 to 100%, as determined by MegAlign. Further analysis revealed that the novel mutant strains exhibited characteristic amino acid sites are 252I, 254 N, 262Y, 299S and 318D. Phylogenetic analysis of the IBDV isolates and reference strains from South China demonstrated that the novel mutant strain has diverged from previously prevalent mutant strains, such as Variant E and GLS, forming a distinct lineage. This finding implies that the high mutation rate of IBDV may compromise vaccine efficacy and pose new challenges for the prevention and control of IBDV in poultry production.

## 1 Introduction

IBD is an acute and highly contagious immunosuppressive disease in chickens caused by the IBDV (1). This virus significantly impairs the host immune system, leading to reduced immune competence and increased susceptibility to other pathogens (2–5). IBDV is classified into two serotypes: Serotype I and Serotype II. Serotype I is pathogenic to chickens and is the primary cause of infections in poultry flocks (6). Notably, there are antigenic differences among the various subtypes of Serotype I, which can lead to variations in the efficacy of vaccine-induced immune protection (7–9). In the past 30 years, under favorable feeding and management conditions and the widespread use of effective vaccines, the virulent strain has been well controlled in China. However, the widespread use of vaccines has triggered high immune selection pressure, promoting the development of genetic diversity in circulating viruses (10). In the past five years, a new mutant strain has emerged in China and has rapidly spread throughout the country, becoming the predominant circulating strain (11, 12). The clinical signs of the new variant strain are not always apparent, but it can cause bursal atrophy, leading to severe immunosuppression and a decline in production performance, posing a new threat to the healthy development of China’s poultry industry (13).

IBDV is a non-enveloped virus with a single capsid exhibiting icosahedral symmetry (14). It belongs to the double-stranded RNA virus family, specifically the genus Avibirnavirus (1). The IBDV genome consists of two double-stranded RNA fragments, A and B. Fragment A is approximately 3.2 kb in length and contains two open reading frames (ORFs). ORF1 encodes the non-structural protein VP5, while ORF2 encodes a polyprotein precursor. This precursor is cleaved by the viral serine protease VP4 into mature viral proteins, including the structural proteins VP2 and VP3, as well as the non-structural protein VP4. Fragment B is approximately 2.8 kb in length and contains a single ORF, which encodes the structural protein VP1 (15). This bipartite structure of IBDV fragments A and B significantly increases the possibility of genome recombination (16, 17). As a result, new variants of IBDV emerge continuously, presenting substantial challenges for the control and prevention of IBD (10, 18).

The VP2 accounts for 51% of the total virion protein and is the sole component of the IBDV outer shell. The VP2 forms three major domains by folding, namely, the basement domain formed by conserved amino acids, the coat domain, and the protrusion domain (19–21). The protrusion domain is formed by the hypervariable region (HVR) of VP2, covering the amino acid 206 to 350 positions, and it is widely believed that the differences between IBDV strains are mainly concentrated in the VP2 HVR (22, 23). In addition, there are 2 hydrophilic regions (aa 210 ~ 225 in the first hydrophilic region and aa 314 ~ 324 in the second hydrophilic region) and 1 heptad region (aa 326 ~ 332) (24). The first hydrophilic region that stabilizes the IBDV conformation, the structure formed by the second hydrophilic region can specifically bind to the neutralizing antibodies, and mutations in the site in the second hydrophilic region may induce antigenic and virulence variation. The heptapeptide region is correlated with the virulence of the virion (25, 26). The two small hydrophilic regions (aa 247 ~ 254 and aa 281 ~ 292) in the VP2 HVR were associated with the virulence and surface antigen of the IBDV strain (17).

The tip of the VP2 HVR is located in the most lateral part of the virion and contains four Loop ring structures, all of which play important roles. P_BC_ (aa 204 ~ 236) covers the first hydrophilic region, and the amino acid at position 222 is related to the replication efficiency of IBDV. The amino acid mutation in this position can change the antigenicity of the strain and lead to immune escape (27). P_HI_ covers the second hydrophilic region with cellular receptor binding sites. Both ring structures contain neutralizing antigen epitopes and, because of their high degree of serum specificity, can be used to distinguish classical, variant, and serotype II strains (28). P_DE_ (aa 240 ~ 265) and PFG (aa 270 ~ 293) were associated with antigenic drift, cytotropism, and virulence of the strain (29). It have found that the two-site mutation Q253H / A284T can adapt the vvIBDV strain not adapted to *in vitro* cell culture and significantly reduce its lethality, and the mortality rate can be reduced from 60% to 0 (30). Therefore, mutations in this regional locus may alter tissue tropism as well as affect virulence, providing a reference for vaccine development.

Molecular epidemiological studies of IBDV have primarily focused on two aspects: molecular characteristics and genetic evolution. Molecular characteristics can serve as a reference for pathogen typing, while genetic evolution studies are of great significance for understanding the evolutionary trends of strains. Currently, research on molecular characteristics primarily focuses on the hypervariable region of *vp2* gene. It’s the protective antigen gene of IBDV and is relatively conserved among very-virulent, classical, and variant strains. Amino acid mutations within the hypervariable region are associated with antigenic differences between strains, with unique amino acid sites in this region playing a critical role (31, 32). These epitopes play a critical role in the host’s antiviral immune response (33, 34). Therefore, genetic and evolutionary analyses of the *vp2* gene can provide valuable insights into the epidemiological trends of IBD. This information is essential for guiding the development of effective IBD vaccines in clinical settings.

Therefore, in this study, an epidemiological investigation was conducted in South China in 2023 to isolate and analyze the genetic evolution of IBDV strains. This investigation aimed to elucidate the molecular epidemiological characteristics of IBDV and comprehensively evaluate the epidemic features of circulating strains. The findings provide valuable insights for the effective prevention and control of infectious bursal disease, as well as for the research and development of vaccines.

## 2 Materials and methods

### 2.1 Ethics statement

This study was approved by the Animal Care Committee of South China Agricultural University (approval ID: SYXK-2019-0136). All study procedures and animal care activities were conducted per the recommendations in the Guide for the Care and Use of Laboratory Animals of the Ministry of Science and Technology of the People’s Republic of China.

### 2.2 Animals

For the experimental procedures, 10- to 12-day-old SPF (Specific Pathogen-Free) embryoated chicken eggs were procured from the SPF Experimental Animal Center of Guangdong Xinxing Dahuanong Poultry Egg Co., Ltd.

### 2.3 Clinical samples

During 2023, outbreaks characterized by atrophy of the bursa of Fabricius occurred in commercial poultry farms in Guangxi, Guangdong, and Fujian, China. A total of 60 clinical samples (including spleens and bursae) were collected from diseased chickens across six commercial farms (includeing two farms in Guangdong, two farms in Guangxi and two farms in Fujian). The date of age of sick chickens is 31 to 63 days. All of the chickens were vaccinated with a live-attenuated W2512 vaccine. All samples, labeled with collection dates and regional locations, were transported to the laboratory on frozen carbon dioxide for processing and analysis.

### 2.4 Virus identification and isolation

The virus isolation procedure was refer to a previously published method (35). Briefly, sixty diseased tissues (including spleen and bursa) were minced and homogenized in a solution containing 5,000 U/mL of penicillin and streptomycin. The homogenate was subjected to three cycles of freeze-thawing at −80°C to disrupt the tissue and release the virus. Subsequently, the mixture was centrifuged at 10,000 rpm for 5 min at 4°C to separate the supernatant, which was then filtered through a 0.22 μm filter to remove any remaining cellular debris. The filtered supernatant was inoculated onto the chorioallantoic membrane of 10- to 12-day-old SPF chicken embryos. Two PBS samples were used as negative controls. Each inoculation consisted of 0.2 mL, sealed with paraffin, and incubated in a 37°C incubator. The embryoated chicken eggs that died within 24 h were discarded. Starting from the first day post-inoculation, dead chicken embryos were collected daily and stored at 4°C. After 5 days of inoculation, the allantoic membranes and a portion of the allantoic fluid were collected from all chicken embryos. The collected materials were mixed with a double antibiotic solution at a ratio of 1:3, homogenized by grinding, and then frozen. The homogenate was subsequently used to inoculate SPF chicken embryos. This process was repeated for three generations (F3), and the final generation’s chorioallantoic membranes and allantoic fluid were homogenized to prepare the viral stock. The embryoated chicken eggs were harvested, and both the chorioallantoic membrane and a portion of the allantoic fluid were collected for RNA extraction and virus detection.

### 2.5 Sequencing and analysis

RNA extraction was performed according to the instructions of the Magen viral DNA/RNA extraction kit. The extracted RNA was tested using the already published universal detection primers for IBDV (IBDV-F: 5’-GCCGATGATTACCAATTCTCATC-3′, IBDV-R: 5’-CCGGATTATGTCTTTGAAGC-3′) (35). And synthesized by Shanghai Biotech Co., Ltd. The 31 isolates were subjected to reverse-transcription polymerase chain reaction (Reverse Transcription-Polymerase Chain Reaction, RT–PCR) with a one-step RT–PCR kit, and the PCR reaction procedure was: 50°C 30 min; 94°C 4 min; 94°C 30s, 55°C 45 s, 72°C 40s, 32 cycles; 72°C 10 min; 4°C terminated. The 7 μL PCR amplification products were detected using 1% agarose gel electrophoresis, and then the remaining PCR products were sent to Guangzhou BGI Technology Corporation for Sanger sequencing.

The measured sequences of the hypervariable region of the *vp2* gene were assembled using SeqMan in DNASTAR. The sequences of the *vp2* gene were analyzed using the ClustalW method in MEGA 7.0, and then the neighbor-joining method was employed to conduct phylogenetic analysis of the *vp2* gene and construct the evolutionary tree. Subsequently, the nucleotide sequence identity of the *vp2* gene was analyzed using MegAlign (Table 1).

**Table 1:** Reference strain sequence.

Strains	Origin	Phenotype	Accession No.
IM	USA	Classic	AY029166
2,512	USA	Classic	DQ355819
Variant E	USA	Variant	AF133904
GLS	USA	Variant	AF093794
Hb06v	China	Novel variant	MW795725
SHG19	China	Novel variant	MN393076
SHG352	China	Novel variant	MT179720
SHG358	China	Novel variant	MT179721
OKYM	Netherlands	Very virulent	D49706
HK46	China	Very virulent	AF092943
D6948	Netherlands	Very virulent	DQ646405
BD399	Bangladesh	Very virulent	AF362776
Gx	China	HLJ0504-like	AY444873
HLJ0504	China	HLJ0504-like	GQ451330
CU-1	China	Attenuated	X16107
JD 1	China	Attenuated	AF321055
B87	China	Attenuated	DQ906921
Gt	China	Attenuated	DQ403248
CT	France	Attenuated	AJ310185
D78	USA	Attenuated	AF499929
OH	Canada	Serotype II	U30818

## 3 Results

### 3.1 Clinical features of the diseased chickens

**Figure 1:**
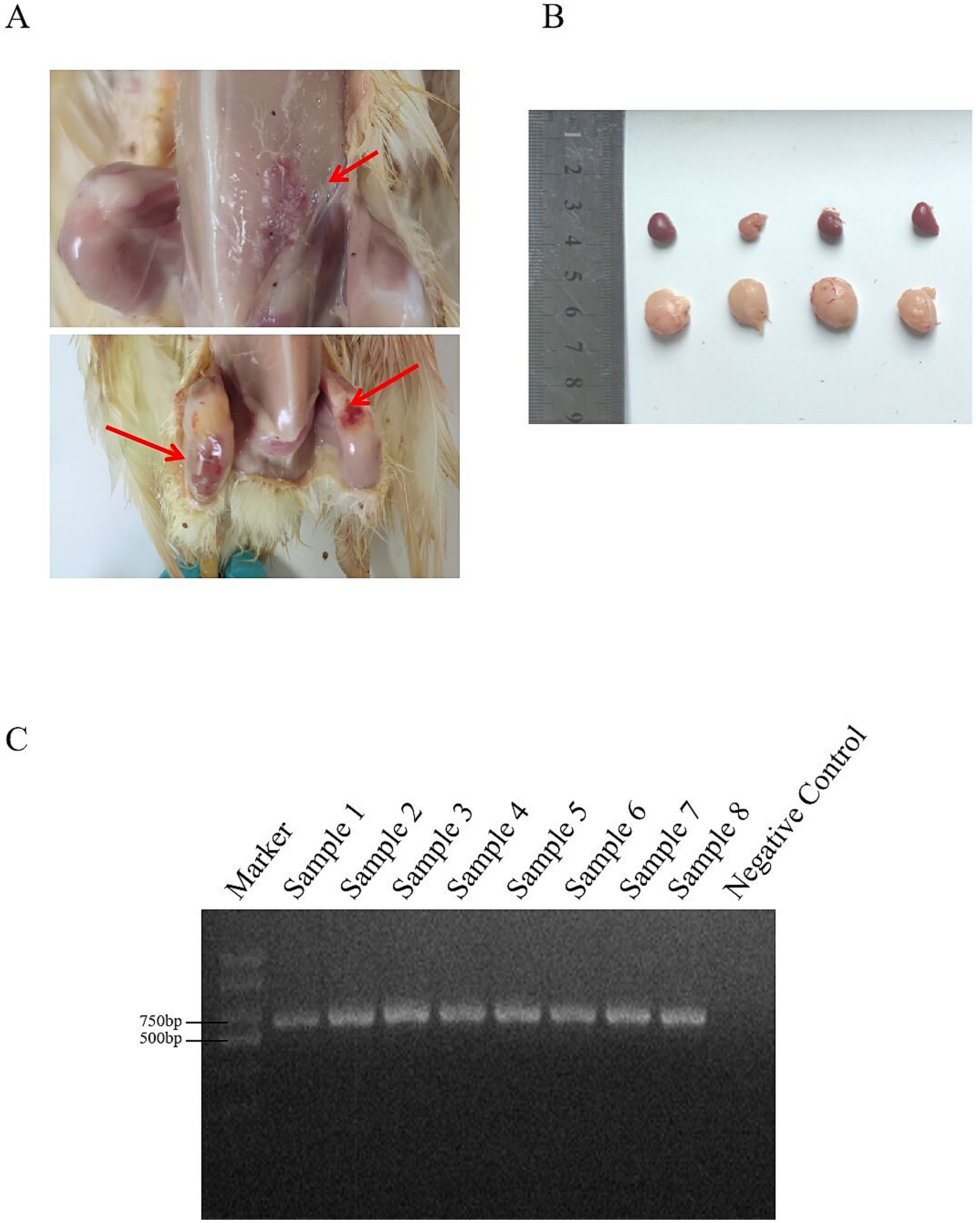
**(A)** The lesions were haemorrhage of leg muscle and pectoral muscle of IBDV-infection chickens. **(B)** The lesions of the bursa of Fabricius and spleen. **(C)** PCR amplification of IBDV.

In 2023, our laboratory received a total of 60 suspected IBDV-infected samples from three provinces in China. The primary clinical symptoms observed in the affected chickens included depression, drooping heads, and sparse feathers. Upon necropsy, the findings included leg hemorrhages, bursal enlargement, and splenic enlargement (Figures 1A,B). The diseased material was tested using the VP2 universal primer with a band size of 714 bp, meeting the expected band size (Figure 1C). A total of 31 positive samples were detected, resulting in a positivity rate of 51.67%.

### 3.2 Phylogenetic tree analysis of *vp2* gene of IBDV isolates

**Figure 2:**
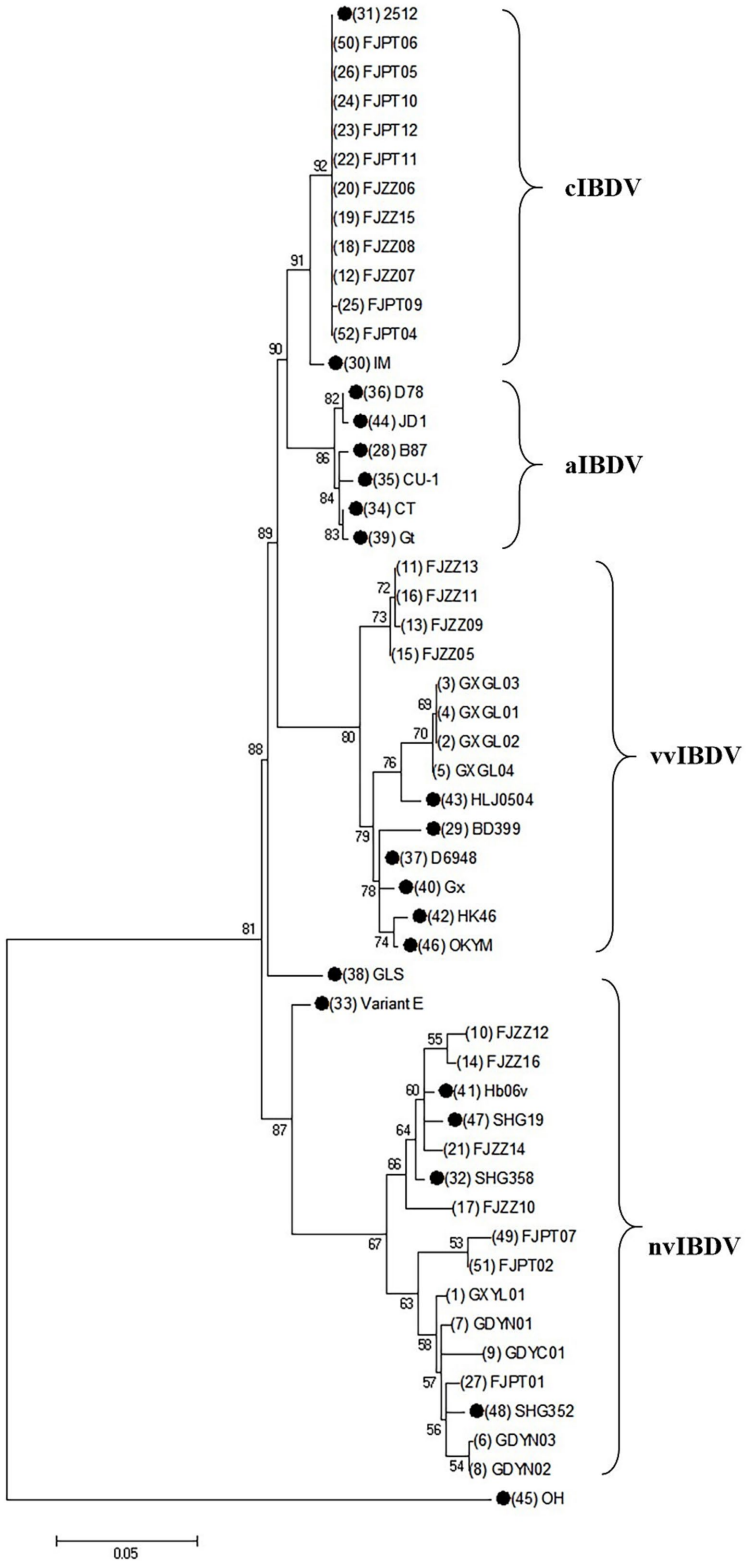
Phylogenic analysis based on the nucleotide sequences of *vp2* gene. A phylogenic tree was constructed using the neighbor-joining method from phylogenetic distances calculated using MEGA 7.0. Bootstrap values obtained from 1,000 replicates are shown at the major nodes. The reference strains in this study are indicated by solid dots.

The nucleotide sequences of the *vp2* gene of IBDV isolates have been deposited in the GenBank database (Table 2). Phylogenetic analysis at the nucleotide level of *vp2* gene of the IBDV isolates, together with reference sequences was conducted. The results showed that the isolates belong to Serotype I and can be classified into three genotypes: very virulent, classical, and novel variants. 12 isolates, including FJZZ10, were identified as novel variants, accounting for 38.7% of the total isolates. Phylogenetic analysis revealed that these novel variants have diverged from the earlier circulating variants, such as Variant E and GLS, and have independently formed a distinct new clade. 11 isolates were identified as classical strains, representing 35.4% of the total isolates. These isolates exhibited a high degree of genetic identity to the reference strain 2512. 8 isolates, including FJZZ05, were identified as very virulent strains, constituting 25.8% of the total isolates. Based on these results, the predominant circulating strains in the sampled regions are the classical strains (Figure 2).

**Table 2:** Information on IBDV isolates and *vp2* gene by region.

Isolate	Isolated region	Phenotype	Accession No.
FJPT01	FUJIAN	Novel variant	PQ609832
FJPT02	FUJIAN	Novel variant	PQ609833
FJPT04	FUJIAN	Classic	PQ609835
FJPT05	FUJIAN	Classic	PQ609836
FJPT06	FUJIAN	Classic	PQ609837
FJPT07	FUJIAN	Novel variant	PQ609838
FJPT09	FUJIAN	Classic	PQ609840
FJPT10	FUJIAN	Classic	PQ609841
FJPT11	FUJIAN	Classic	PQ609842
FJPT12	FUJIAN	Classic	PQ609843
FJZZ05	FUJIAN	Very virulent	PQ609805
FJZZ06	FUJIAN	Classic	PQ609806
FJZZ07	FUJIAN	Classic	PQ609807
FJZZ08	FUJIAN	Classic	PQ609808
FJZZ09	FUJIAN	Very virulent	PQ609809
FJZZ10	FUJIAN	Novel variant	PQ609810
FJZZ11	FUJIAN	Very virulent	PQ609811
FJZZ12	FUJIAN	Novel variant	PQ609812
FJZZ13	FUJIAN	Very virulent	PQ609813
FJZZ14	FUJIAN	Novel variant	PQ609814
FJZZ15	FUJIAN	Classic	PQ609815
FJZZ16	FUJIAN	Novel variant	PQ609816
GDYC01	GUANGDONG	Novel variant	PQ609821
GDYN01	GUANGDONG	Novel variant	PQ609817
GDYN02	GUANGDONG	Novel variant	PQ609818
GDYN03	GUANGDONG	Novel variant	PQ609819
GXGL01	GUANGXI	Very virulent	PQ609899
GXGL02	GUANGXI	Very virulent	PQ609900
GXGL03	GUANGXI	Very virulent	PQ609901
GXGL04	GUANGXI	Very virulent	PQ609902
GXYL01	GUANGXI	Novel variant	PQ609820

### 3.3 Identity analysis of the *vp2* gene in IBDV isolates

Among the 31 IBDV isolates obtained in this study, the nucleotide sequence fragment length was 714 base pairs (bp) (spanning positions 654 bp to 1,368 bp), and encode approximately 238 amino acids. The nucleotide sequences of the hypervariable region of the *vp2* gene were aligned with each other and to the reference sequence. The results revealed that the nucleotide sequence identity of the *vp2* gene ranged from 90.7 to 100% among the 31 isolates. The identity between the isolated classical strains and the reference sequence 2512 was 99.8 to 100%. The identity between the isolated novel variant strains and the reference strains SHG352 and SHG19 was 94.5 to 99.7%, and the identity between the very virulent strains and the reference sequence HLJ0504 was 95.6 to 100% (Supplementary Figure S1). The alignment of the amino acid sequence in the hypervariable region of VP2 shows that 11 classical strains showed 100% identity with reference strain 2512, 12 novel variants strains showed 98.1 to 100% identity with reference strains SHG352 and SHG19, and 8 very virulent strains showed 98.1 to 100% identity with reference strain HLJ0504 (Supplementary Figure S2).

### 3.4 Molecular characteristic of the *vp2* gene of IBDV isolates

**Figure 3:**
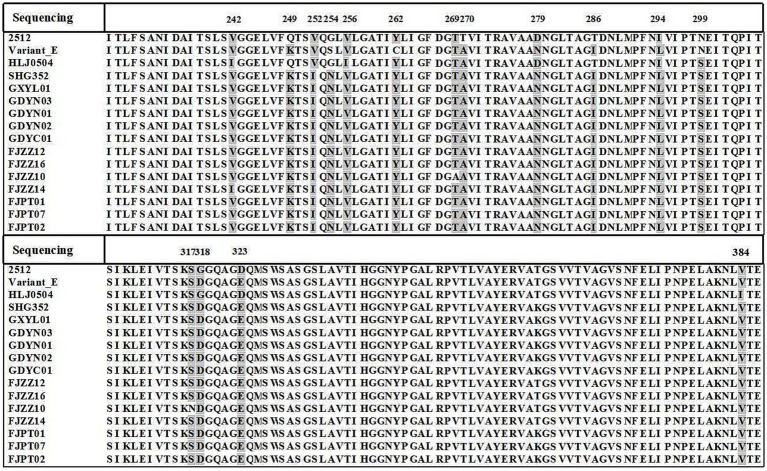
Amino acid sequence alignment of *vp2* gene. Amino acid alignment of 12 novel variants with variant E, HLJ0504, 2512, and SHG352 using MegAlign. All variant residues within the sequences are highlighted against a background. The amino acid coordinates are provided at the top.

The characteristic amino acid alignment based on the *vp2* hypervariable region showed that the 12 novel variants had different amino acid residues, including 252I, 254 N, 262Y and 299S. And, amino acid site mutations such as 270A and 317S. Furthermore, alignment analysis of the amino acid sequence with virulent strain HLJ0504 revealed a difference of 12 amino acid sites between the isolates and the reference sequence, which may be associated to strain variation. Notably, the 12 isolates had the characteristic amino acid sites of the virulent strains: 299S. And compared with the current novel variant SHG352, isolate FJZZ14 mutation at site 242 (V to I), FJZZ10 mutated at 317 (S to N) and 269 (T to A). In conclusion, the amino acid sequence alignment of the *vp2* hypervariable region showed that the characteristic amino acid sites are 252I, 254 N, 262Y, 299S and 318D, indicating that the 12 novel variants have new characteristics and may have the characteristics of virulent strains (Figure 3).

## 4 Discussion

Infectious bursal disease (IBD) is one of the diseases that cannot be ignored in poultry. Its prevalence in China can be traced back to 1979 (36). Among them, very virulent strains have attracted significant attention due to their high incidence, high mortality, and ability to cause severe immunosuppression (16, 37). These very virulent strains pose a serious threat to the healthy breeding of China’s poultry industry, causing substantial economic losses. However, with the modernization of China’s poultry farming practices and effective vaccination programs, outbreaks of very virulent strains have been effectively controlled.

The South China region of China is home to several major poultry-raising provinces. In the past few years, the predominant strains of IBDV circulating in South China were highly virulent or very virulent strains (38, 39). However, in recent years, novel variant strains have been successively detected in South China. These novel variant strains do not cause mortality in chicken flocks but can lead to severe immunosuppression (40, 41). Reports have indicated that the currently used commercial vaccines still fail to prevent the damage of these novel variant strains to the bursa of Fabricius (42). This suggests that commercial IBDV vaccines targeting very virulent strains cannot protect chicken flocks from the novel variant strains, resulting in vaccination failure.

To understand the epidemiological trends of IBD in South China, this study selected three regions: Guangdong, Guangxi, and Fujian for epidemiological detection. A total of 31 strains were isolated, including 12 novel variants, 8 very virulent strains, and 11 classical strains. This indicates that multiple types of strains coexist, and mixed infections of novel variant strains, very virulent strains, and classical strains are gradually becoming the main epidemiological trend in South China.

In this study, the clinical samples were collected from diseased chickens aged 31 to 63 days in six poultry farms across three provinces in southern China (including Guangdong, Guangxi and Fujian). All the chickens vaccinated with attenuated live vaccine at 10-days-old. Therefore, we detected the vaccine strain W2512 in the field. The novel variant strains were isolated in two flocks in Guangdong province, suggesting that the attenuated live vaccine provides partial protection against novel variants. Several reports also confirmed the partial protection of commercial vaccines against novel variants (43). Our data further highlight the urgent need to reevaluate vaccine formulations, particularly in regions where novel variants are endemic.

Notably, alignment analysis of the *vp2* HVR in 12 novel variants revealed conserved residues 286I and 318D, consistent with previously reported variant-specific signatures. In particular, not only distinguishes these novel variants from historical lineages (e.g., early Variant E) but also emerges as a potential key site driving viral evolution. Phylogenetic reconstruction based on *vp2* HVR sequences demonstrated that the 12 novel variants diverged significantly from earlier strains (e.g., Variant E and GLS) circulating in the United States, forming a distinct clade with multiple sublineages. Furthermore, among eight newly isolated strains (GXGL01–04 and FJZZ09–13), four clustered with the reference virulent strain HLJ0504, while the remaining four (FJZZ09, FJZZ11, and FJZZ13) constituted an independent subclade. This genetic diversification underscores the ongoing evolution of IBDV *vp2* in southern China, posing challenges for regional disease control strategies. These findings highlight two critical implications: The coexistence of variant-specific (286I/318D) and virulence-associated (299S) residues in novel variants suggests a convergent evolutionary trajectory, potentially enhancing both immune evasion and pathogenicity. The emergence of multiple subclades within the novel variant group reflects accelerated genomic diversification, likely driven by selective pressures in immunized flocks. Such evolutionary trends necessitate vigilant surveillance of *vp2* polymorphisms to update vaccine formulations and refine diagnostic tools. Future studies should explore the structural and functional consequences of 299S and other critical residues using reverse genetics, as well as assess cross-protective efficacy of current vaccines against these evolving lineages.

In conclusion, this study isolated and identified 31 IBDV isolates, including 8 very virulent strains, 11 classical strains, and 12 novel variants. Further analysis revealed that the novel variant strains exhibited characteristic amino acid sites are 252I, 254 N, 262Y, 299S and 318D, suggesting that these isolates possess traits of both mutant and virulent phenotypes. Phylogenetic analysis of IBDV isolates and reference strains from South China demonstrated that the novel variant strains were distinct from previously reported strains, such as variant E and GLS, and formed a unique evolutionary lineage. These findings indicate that the high mutation rate of IBDV may compromise vaccine efficacy and present new challenges for the prevention and control of IBDV in poultry production.

## Data Availability

The datasets presented in this study can be found in online repositories. The names of the repository/repositories and accession number(s) can be found in the article/Supplementary material.

## Supplementary material

SUPPLEMENTARY FIGURE S1 Alignment of nucleotide sequence identity in the hypervariable region of the vp 2 gene.

SUPPLEMENTARY FIGURE S2 Alignment of amino acid sequence identity in the hypervariable region of the vp 2 gene.
