# Supplementary figures and images for: Phylogenetic analysis of vp2 gene of the infectious bursal disease virus in South China during 2023
Source: Front Vet Sci. 2025 Apr 15;12:1575407. doi: 10.3389/fvets.2025.1575407 (PMC12037624; doi:10.3389/fvets.2025.1575407)

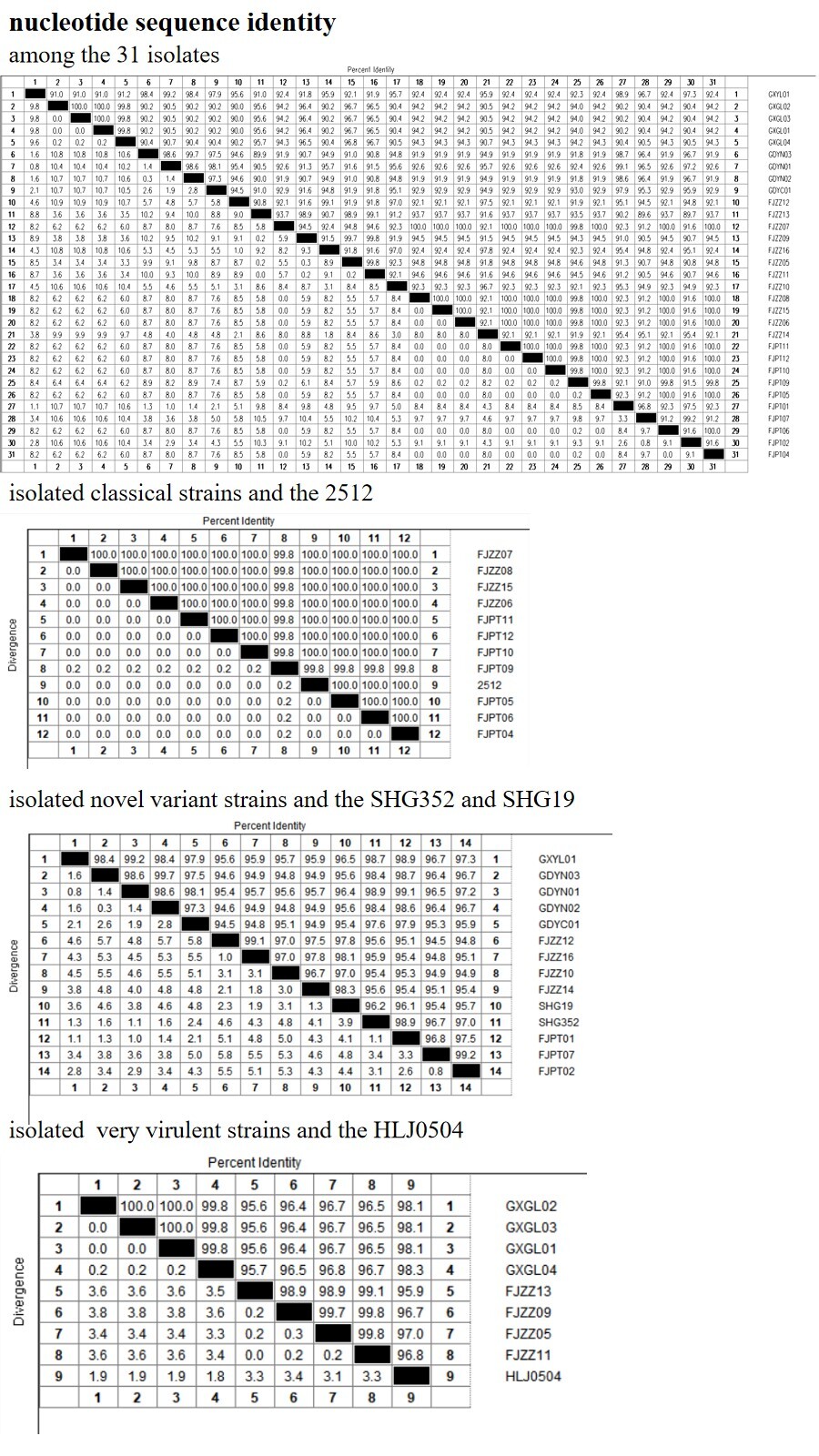

Supplement: SUPPLEMENTARY FIGURE S1 — Alignment of nucleotide sequence identity in the hypervariable region of the vp 2 gene. [file Image_1.jpg]

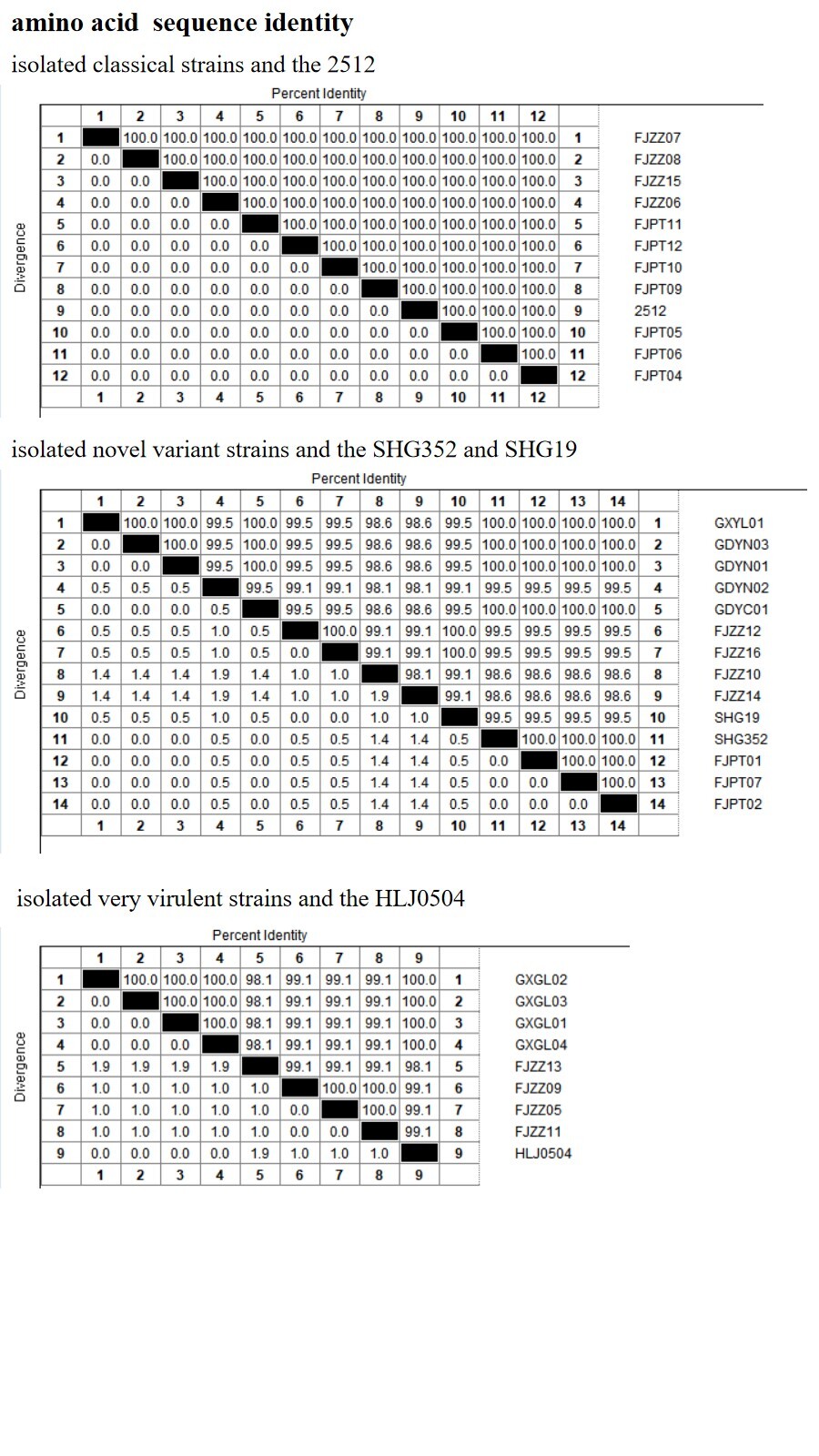

Supplement: SUPPLEMENTARY FIGURE S2 — Alignment of amino acid sequence identity in the hypervariable region of the vp 2 gene. [file Image_2.jpg]
